# Prime–boost vaccination with plasmid and adenovirus gene vaccines control HER2/*neu*^+ ^metastatic breast cancer in mice

**DOI:** 10.1186/bcr1199

**Published:** 2005-05-23

**Authors:** Xiaoyan Wang, Jian-Ping Wang, Xiao-Mei Rao, Janet E Price, Heshan S Zhou, Lawrence B Lachman

**Affiliations:** 1 Department of Experimental Therapeutics, The University of Texas MD Anderson Cancer Center, Houston, TX, USA; 2 Program in Immunology, The University of Texas Graduate School of Biomedical Sciences at Houston, Houston, TX, USA; 3 Department of Medicine, University of Louisville, Louisville, KY, USA; 4 Department of Cancer Biology, The University of Texas MD Anderson Cancer Center, Houston, TX, USA

## Abstract

**Introduction:**

Once metastasis has occurred, the possibility of completely curing breast cancer is unlikely, particularly for the 30 to 40% of cancers overexpressing the gene for HER2/*neu*. A vaccine targeting p185, the protein product of the HER2/*neu *gene, could have therapeutic application by controlling the growth and metastasis of highly aggressive HER2/*neu*^+ ^cells. The purpose of this study was to determine the effectiveness of two gene vaccines targeting HER2/*neu *in preventive and therapeutic tumor models.

**Methods:**

The mouse breast cancer cell line A2L2, which expresses the gene for rat HER2/*neu *and hence p185, was injected into the mammary fat pad of mice as a model of solid tumor growth or was injected intravenously as a model of lung metastasis. SINCP-*neu*, a plasmid containing Sindbis virus genes and the gene for rat HER2/*neu*, and Adeno-*neu*, an E1,E2a-deleted adenovirus also containing the gene for rat HER2/*neu*, were tested as preventive and therapeutic vaccines.

**Results:**

Vaccination with SINCP-*neu *or Adeno-*neu *before tumor challenge with A2L2 cells significantly inhibited the growth of the cells injected into the mammary fat or intravenously. Vaccination 2 days after tumor challenge with either vaccine was ineffective in both tumor models. However, therapeutic vaccination in a prime–boost protocol with SINCP-*neu *followed by Adeno-*neu *significantly prolonged the overall survival rate of mice injected intravenously with the tumor cells. Naive mice vaccinated using the same prime–boost protocol demonstrated a strong serum immunoglobulin G response and p185-specific cellular immunity, as shown by the results of ELISPOT (enzyme-linked immunospot) analysis for IFNγ.

**Conclusion:**

We report herein that vaccination of mice with a plasmid gene vaccine and an adenovirus gene vaccine, each containing the gene for HER2/*neu*, prevented growth of a HER2/*neu*-expressing breast cancer cell line injected into the mammary fat pad or intravenously. Sequential administration of the vaccines in a prime–boost protocol was therapeutically effective when tumor cells were injected intravenously before the vaccination. The vaccines induced high levels of both cellular and humoral immunity as determined by *in vitro *assessment. These findings indicate that clinical evaluation of these vaccines, particularly when used sequentially in a prime–boost protocol, is justified.

## Introduction

Once metastasis has occurred, the possibility of completely curing breast cancer is unlikely [1], particularly for the 30 to 40% of cancers overexpressing the gene for HER2/*neu *[2]. A vaccine targeting HER2/*neu *could have considerable preventive and therapeutic application by controlling the growth and metastasis of highly aggressive HER2/*neu*^+ ^tumor cells [3,4]. Gene vaccines, which are bacterial expression plasmids encoding the DNA sequence for tumor antigens, have induced strong antitumor immunity in animals [5]. Although gene vaccines have shown effectiveness in clinical trials [6-13], only a few trials have been completed in oncology patients, and the results have been mixed [14-19]. However, it was recently demonstrated that a gene vaccine for prostate-specific antigen broke immunologic tolerance and induced cellular immunity [20]. Immunotherapy for cancer using gene vaccines is in its infancy, and the development of new approaches and techniques is anticipated [21-24].

The fields of gene therapy and gene vaccines have recently converged, as shown by the use of identical delivery systems for both purposes [25]. Alphaviruses such as the Sindbis, Semliki Forest, and Venezuelan equine encephalitis viruses may be used for both gene therapy and gene vaccines [26-28]. We [29] and others [30] have shown that plasmids containing Sindbis virus genes induce excellent antitumor immunity in murine tumor models. Adenoviruses, the workhorse of gene replacement therapy, are quickly moving to the forefront as gene vaccine vectors [31-35]. Unlike alphaviruses, which contain self-replicating RNA [36], adenoviruses contain DNA as their genetic material. For both families of viruses, essential genes for replication or packaging are deleted or mutated to ensure the safety of the gene therapy or gene vaccine vector.

Although animal models of tumor growth are limited in their ability to represent clinical cancer, models can provide valuable information about drug candidates. We have used both preventive and therapeutic murine tumor models to evaluate the effectiveness of two gene vaccines. Our results demonstrated that each gene vaccine was effective in prevention models, but neither was effective when used in a therapeutic model. However, prime–boost vaccination with SINCP-*neu *followed by Adeno-*neu *significantly prolonged the overall survival rate when used therapeutically in a murine model of breast cancer metastasis. This finding indicates that effective vaccine immunotherapy may require treatment with at least two gene vaccines delivered in a precise order.

## Materials and methods

### Tumor cell line and reagents

The A2L2 cell line, which expresses high levels of rat HER2/*neu*, was derived from the mouse tumor cell line 66.3 in our laboratory [29]. The A2L2 cell line has expressed high levels of HER2/*neu *for more than 5 years and consistently induces tumors in BALB/c mice when injected into a mammary fat pad or intravenously (i.v.). The line was maintained in Eagle's minimal essential medium containing 5% fetal bovine serum, sodium pyruvate, nonessential amino acids, L-glutamine, and vitamins (Gibco, Carlsbad, CA, USA). The monolayer cultures were subdivided at approximately 75% confluence by treatment for 1 to 3 min with 0.25% trypsin and 0.02% ethylenediaminetetraacetic acid (EDTA) at 37°C.

### SINCP-*neu *and Adeno-*neu *gene vaccines

The SINCP plasmid, which contains Sindbis virus genes, was provided by Dr John Polo (Chiron, Emeryville, CA, USA). SINCP is nearly identical to the ELVIS plasmid we previously tested [29] except for an internal ribosome entry site on the 5' side of the insertion point for an antigen gene. The gene for rat *neu *was excised from pSV2-*neu *and inserted into SINCP by standard recombinant DNA techniques [29]. The correct insertion of the complete *neu *gene was confirmed by sequence analysis. The E1,E2a-deleted adenovirus (Adeno) has been described in detail [37,38]. The gene for rat *neu *was excised from pSV2-*neu *and incorporated into Adeno as previously described [38].

### Flow cytometric analysis

To evaluate the level of immunoglobulin G (IgG) in the serum of vaccinated mice, we incubated A2L2 cells for 1 hour at 37°C with either immune serum or control serum diluted 1:100 in PBS, pH 7.2. Fluorescein-isothiocyanate-labeled (FITC-labeled) goat antimouse IgG (Pharmingen, San Diego, CA, USA) diluted 1:1000 in PBS was added to the cells, and incubation continued for 1 hour at 37°C. The cells were washed three times in PBS and analyzed by flow cytometry using an EPICS Profile Analyzer (Coulter, Hialeah, FL, USA). As a positive control, A2L2 cells were stained with a 1:1000 dilution of a polyclonal rabbit antibody against p185 (sc-284; Santa Cruz Biotechnology, Santa Cruz, CA, USA) and an FITC-labeled goat anti-rabbit IgG.

### Mice

Female BALB/c mice 6 to 8 weeks old were obtained from the National Cancer Institute-Frederick Cancer Research Facility (Frederick, MD, USA). Mice were acclimated for at least 1 week before use. Our small-animal facility is approved by the Association for the Assessment and Accreditation of Laboratory Animal Care (AAALAC).

### Vaccination of mice with SINCP-*neu *and Adeno-*neu*

SINCP-*neu *was prepared in milligram quantities by Aldevron (Fargo, ND, USA). Intramuscular (i.m.) injections of 0.1 ml containing 100 μg of plasmid DNA formulated with 0.25% bupivacaine (Sigma Chemical, St Louis, MO, USA) were administered to the quadriceps using a 24-gauge needle. Adeno-*neu *and adenovirus lacking an inserted gene (Adeno-empty) were suspended in 100 μl of 0.85% saline and injected into the quadriceps using a 24-gauge needle. Blood was collected at intervals from the tail vein, and the serum was separated by centrifugation after incubation at 37°C for 1 hour and overnight refrigeration.

### Mammary fat pad tumor prevention model

We used a mammary fat pad tumor prevention model to assess the effect of vaccination on solid tumor development. A2L2 cells from cultures that had reached 75% confluence were harvested by treatment with 0.25% trypsin and 0.02% EDTA for 1 to 3 min at 37°C. The cells were washed once in serum-containing culture medium and once in PBS. Mice were anesthetized by inhalation of isoflurane using an apparatus developed by veterinarians at the University of Texas MD Anderson Cancer Center [39]. The fur was shaved over the lateral thorax, and a 5-mm-long incision was made to reveal mammary fat pad number 2 [40]. A 0.1-ml sample containing 2.5 × 10^4 ^A2L2 cells in normal saline was injected into the fat pad. The incision was closed with a wound clip. After 7 days, wound clips that had not already fallen off were removed. The mice were then observed daily, and their tumors were measured in perpendicular directions using a caliper. Mice with tumors at least 10 mm in the greater dimension were killed according to our approved Institutional Animal Care and Use protocol. At the end of the experiment, all mice were killed by CO_2 _inhalation, and all tumors were excised and weighed. Examination of the surface of the lungs during necropsy did not reveal tumor nodules.

### Experimental metastasis prevention model

Although a solid tumor develops after injection of A2L2 cells into the mammary fat pad, it is not highly metastatic. The tumor induces moribundity in mice before an appreciable number of lung metastases develop. For this reason, we used an experimental lung metastasis model rather than a spontaneous lung metastasis model to assess the effect of vaccination on lung metastasis. A 0.1-ml sample containing 2.5 × 10^4 ^A2L2 cells in normal saline was injected into the tail vein of each immunized mouse. Thirty days later, the mice were killed by CO_2 _inhalation, and surface lung metastases in each animal were counted.

### ELISPOT analysis of IFNγ production by immune spleen cells

To determine the number of interferon-γ-producing (IFNγ-producing) cells in the spleens of vaccinated mice, we used an IFNγ enzyme-linked immunospot (ELISPOT) technique (kit no. 552569; Pharmingen) and used reagents from Pharmingen whenever possible. Immune spleens were dissected from vaccinated mice and prepared exactly as previously described for tetramer analysis [41]. Wells containing only immune spleen cells served as negative controls, and spleen cells from vaccinated mice cultured with 5 μg/ml concanavalin A (Con A) (Sigma-Aldrich, St Louis, MO, USA) overnight served as a positive control. The finished plates were air-dried overnight at room temperature in the dark and sent to ZellNet Consulting (New York, NY, USA [42]), where the spots were counted automatically using an ImmunoSpot Series I analyzer (BD Bioscience, San Diego, CA, USA). If confluence (overlapping spots) was present in wells, the number of spots in a non-confluent area of that well was determined, and the following equation was used to estimate the total number of spots in each well with confluence: total spot number = spot count+2(spot count × % confluence/ [100% – %confluence]).

### Statistical analysis

Student's *t *test was performed using Prism 4.0 GraphPad software (San Diego, CA, USA).

## Results

### Inhibition of solid tumor growth by vaccination with SINCP-*neu *and Adeno-*neu*

**Figure 1:**
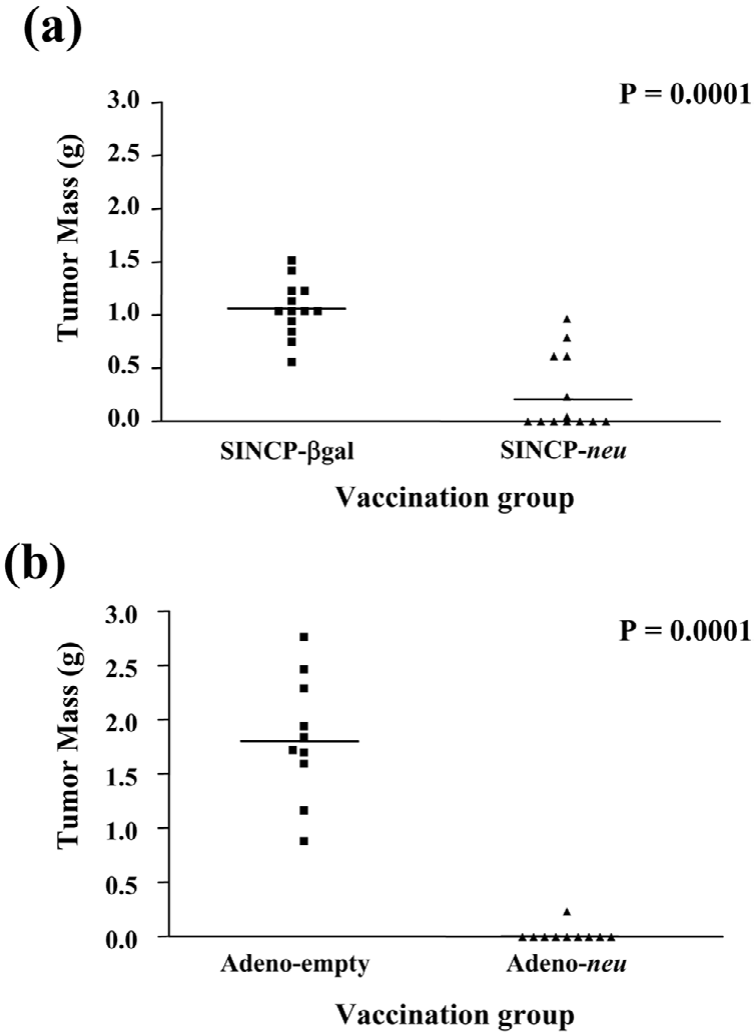
Protection from tumor challenge in the mammary fat pad after vaccination with SINCP or Adeno. Groups of mice were vaccinated once with SINCP-*neu *(a plasmid containing Sindbis virus genes and the gene for rat HER2/*neu*) or SINCP-βgal. **(a) **or with Adeno-*neu *(an E1,E2a-deleted adenovirus also containing the gene for rat HER2/*neu*) or Adeno-empty (adenovirus lacking an inserted gene) **(b)**. Two weeks after vaccination with SINCP and 6 weeks after vaccination with Adeno, the mice were challenged with A2L2 cells injected into a mammary fat pad. Thirty-five days after the tumor challenge, the mice were killed and if a solid tumor was present, the mass of the tumor was determined. Each symbol represents one mouse. Horizontal bars indicate means.

To determine the effectiveness of vaccination on solid tumor growth, two groups of 13 mice each were vaccinated once i.m. in the quadriceps with 100 μg of SINCP-*neu *or SINCP-βgal. Two weeks later, the mice were challenged with A2L2 cells injected into the mammary fat pad. Thirty-five days after the challenge, the mice were killed and if a solid tumor was present, its mass was determined. All of the mice vaccinated with SINCP-βgal developed tumors. In the group vaccinated with SINCP-*neu*, only six mice developed tumors, and the mean mass of these six tumors was significantly less than the mean mass of the tumors in the SINCP-βgal group (0.63 vs 1.02 g; *P *= 0.0186). When we calculated the mean mass for all 13 mice in the SINCP-*neu *group and compared it with the value of the SINCP-βgal group, the difference was significant (0.25 vs 1.02 g; *P *= 0.0001) (Fig. 1a).

Two groups of 10 mice were each vaccinated i.m. in the quadriceps once with 1 × 10^10 ^particles of Adeno-*neu *or Adeno-empty. Six weeks later, the mice were challenged with A2L2 cells injected into the mammary fat pad. Thirty-five days after the challenge, the mice were killed and if a solid tumor was present, its mass was determined. All of the mice vaccinated with Adeno-empty developed tumors, whereas in the group vaccinated with Adeno-*neu *only 1 of 10 mice developed a tumor (mean mass 1.75 vs 0.2 g; *P *= 0.0001) (Fig. 1b).

Our findings show that reduced solid tumor growth is attributable to the induction of cellular immunity. This immunity is antigen specific, because it was absent in mice vaccinated with SINCP-βgal or Adeno-empty, both of which lack the *neu *gene.

### Inhibition of experimental metastasis by vaccination with SINCP-*neu *and Adeno-*neu*

**Figure 2:**
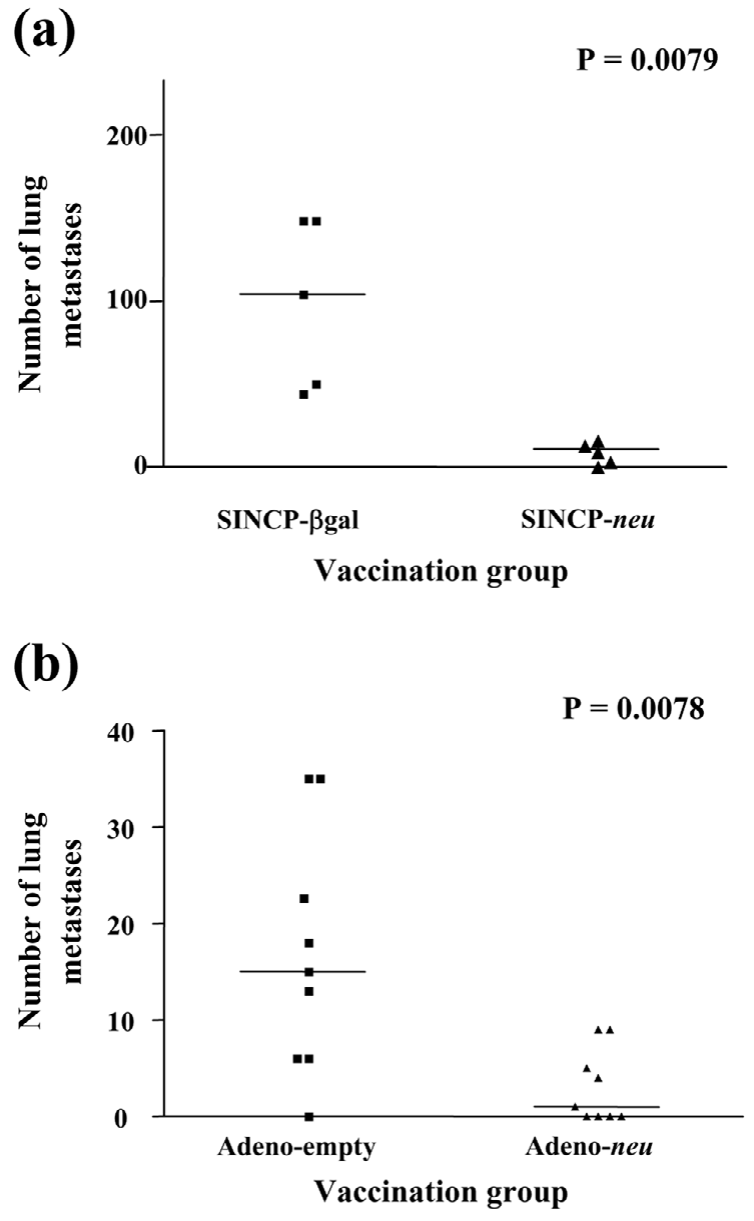
Protection from intravenous tumor challenge after vaccination with SINCP or Adeno. Groups of mice were vaccinated three times at 2-week intervals with SINCP-*neu *(a plasmid containing Sindbis virus genes and the gene for rat HER2/*neu*) or SINCP-βgal. **(a) **or once with Adeno-*neu *(an E1,E2a-deleted adenovirus also containing the gene for rat HER2/*neu*) or Adeno-empty (adenovirus lacking an inserted gene) **(b)**. Two weeks after the final vaccination with SINCP or 6 weeks after vaccination with Adeno, the mice were challenged with A2L2 cells injected into the tail vein. Twenty-one days after the challenge in SINCP-vaccinated animals or 35 days after the challenge in Adeno-vaccinated animals, the mice were killed and the number of tumor nodules on the surfaces of the lungs was counted. Each symbol represents one mouse. Horizontal bars indicate means.

To determine the effectiveness of vaccination on experimental metastasis of A2L2 cells, two groups of five mice each were vaccinated i.m. with 100 μg of SINCP-*neu *or SINCP-βgal three times at 2-week intervals. Two weeks after the third vaccination, the mice were challenged with A2L2 cells injected into the tail vein. Twenty-one days after the challenge, the mice were killed, the lungs removed, and the number of tumor nodules on the surface of the lungs counted with the naked eye. All of the mice in the SINCP-βgal group had more than 25 surface lung nodules, whereas in the SINCP-*neu *group all of the mice had between 1 and 10 nodules (mean number of nodules 105 vs 15; *P *= 0.0079) (Fig. 2a).

Two groups of nine mice each were vaccinated once with Adeno-*neu *or Adeno-empty and 6 weeks later challenged with A2L2 cells injected into the tail vein. Thirty-five days after the challenge, the mice were killed, the lungs removed, and the number of surface lung nodules counted with the naked eye. All but one mouse in the Adeno-empty group had lung nodules, whereas in the Adeno-*neu *group five of nine mice had lung nodules (mean number of nodules 15 vs 1; *P *= 0.0078) (Fig. 2b).

Our results show that inhibition of experimental metastasis is attributable to the induction of cellular immunity. This immunity is antigen specific because it was absent in mice vaccinated with SINCP-βgal or Adeno-empty, both of which lack the *neu *gene.

### *neu*-Specific IgG in the serum of mice vaccinated with SINCP-*neu *or Adeno-*neu*

**Figure 3:**
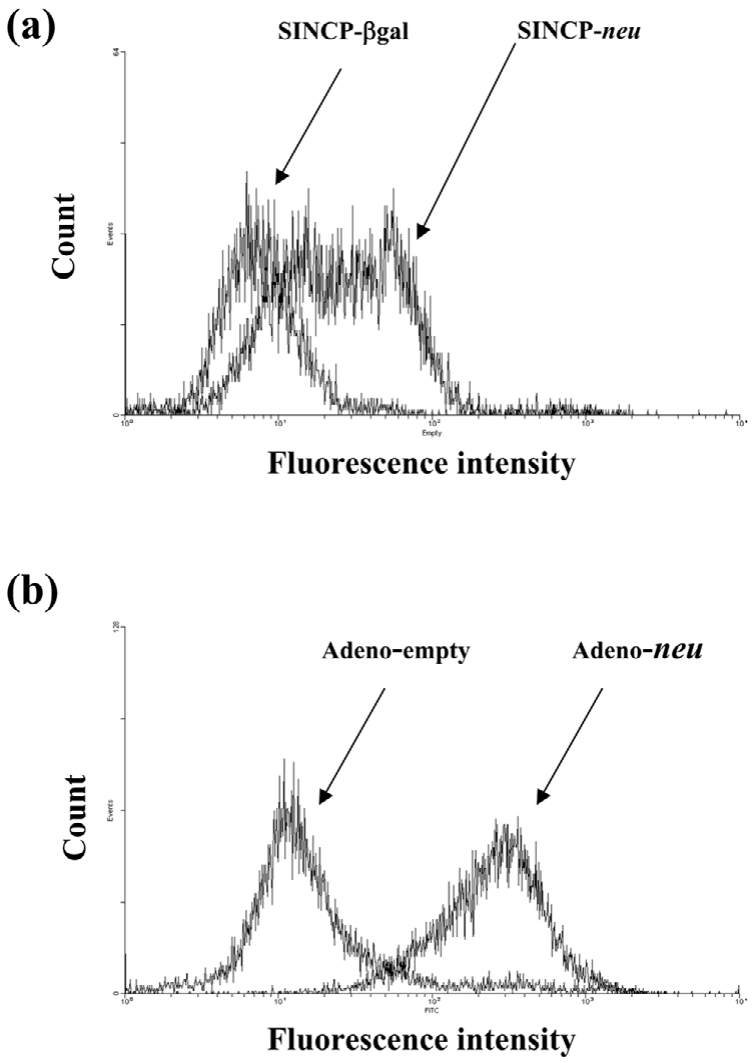
Flow cytometric analysis, using A2L2 cells, of serum from mice vaccinated with SINCP or Adeno. Serum was collected 2 weeks after the third vaccination of mice with SINCP-*neu *(a plasmid containing Sindbis virus genes and the gene for rat HER2/*neu*) or SINCP-βgal. **(a) **or 6 weeks after a single vaccination with Adeno-*neu *(an E1,E2a-deleted adenovirus also containing the gene for rat HER2/*neu*) or Adeno-empty (adenovirus lacking an inserted gene) **(b)**. The immune serum was diluted with PBS (1:100), and fluorescein-isothiocyanate-labeled goat antimouse IgG diluted in PBS (1:1000) was used as the secondary antibody.

To determine if vaccination induced a humoral immune response to p185, immediately before tumor challenge 0.1 ml of blood was collected from the tail vein of each of the mice treated as described for the experimental metastasis model. The sera for each group were pooled and analyzed for the presence of p185-specific IgG by flow cytometry using the A2L2 cell line as described previously [29] and in Materials and methods. Serum from mice vaccinated with SINCP-*neu *had a stronger IgG response to A2L2 cells than that from mice vaccinated with SINCP-βgal (Fig. 3a). Likewise, serum from mice vaccinated with Adeno-*neu *exhibited a stronger IgG response to A2L2 cells than that from mice vaccinated with Adeno-empty (Fig. 3b). These results show that the gene vaccines induce humoral immunity as well as the cellular immunity described above.

### Therapeutic vaccination with SINCP and Adeno

Because SINCP-*neu *and Adeno-*neu *were effective as preventive vaccines delivered before tumor cell challenge, we evaluated both vaccines as therapeutic vaccines delivered after tumor cell challenge. Vaccination conditions were the same as those described for the experimental metastasis model. In both the therapeutic mammary fat pad tumor model and the therapeutic experimental metastasis model, neither vaccine prolonged the overall survival rate of mice when delivered after tumor cell challenge (data not shown). Even when administered as early as 2 days after tumor challenge, neither vaccine was effective.

### Therapeutic vaccination with SINCP and Adeno using a prime–boost protocol

To determine whether the therapeutic effectiveness of the vaccines could be increased by sequential administration, we evaluated a prime–boost protocol in which the mice were primed with a single injection of SINCP 2 days after tumor cell challenge with A2L2 cells and boosted with injections of Adeno 9 and 16 days after the challenge. SINCP was always the prime and Adeno was always the boost, because numerous studies have reported that the most effective protocol is a plasmid followed by a virus [43-47]. All combinations of SINCP-*neu *or SINCP-βgal used as the prime and Adeno-*neu *and Adeno-empty used as the boost were tested in both the therapeutic mammary fat pad tumor model and the therapeutic experimental metastasis model.

**Figure 4:**
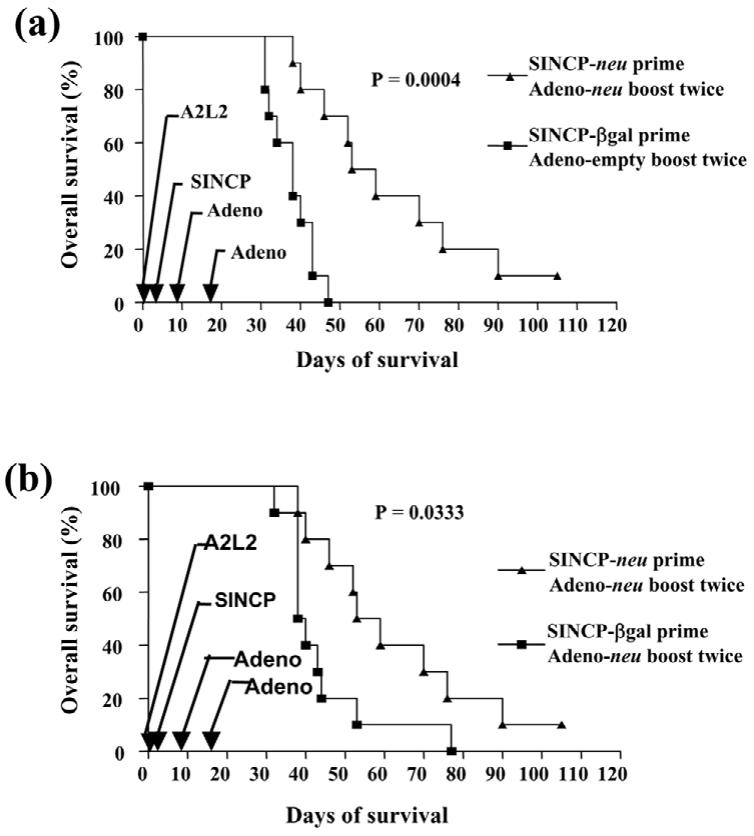
Survival in a therapeutic metastasis model after vaccination with SINCP (prime) and Adeno (boost). Mice were given injections into the tail vein of A2L2 cells on day 0. On day 2, the mice were vaccinated with either SINCP-*neu *(a plasmid containing Sindbis virus genes and the gene for rat HER2/*neu*) or SINCP-βgal and on days 9 and 16 the mice were vaccinated with either Adeno-*neu *(an E1,E2a-deleted adenovirus also containing the gene for rat HER2/*neu*) or Adeno-empty (adenovirus lacking an inserted gene). **(a) **Overall survival of mice vaccinated with SINCP-*neu *and Adeno-*neu *compared with mice vaccinated with SINCP-βgal and Adeno-empty. **(b) **Overall survival of mice vaccinated with SINCP-*neu *and Adeno-*neu *compared with mice vaccinated with SINCP-βgal and Adeno-*neu*. *P *values were determined by a log-rank test of Kaplan–Meier survival curves.

In the therapeutic mammary fat pad tumor model, none of the combinations significantly increased the overall survival rate (data not shown). In the therapeutic experimental metastasis model, only one prime–boost combination was effective: the overall survival rate was significantly higher when SINCP-*neu *was the prime and Adeno-*neu *was the boost (*P *= 0.0004) (Fig. 4a). A comparison of mice vaccinated with SINCP-βgal as the prime showed that the overall survival rate was slightly higher when Adeno-*neu *was the boost than when Adeno-empty was the boost, but that this increase was not as high when *neu *was in both the prime and the boost (Fig. 4b). Thus, priming with SINCP-*neu *is essential for the effect, depends on the presence of the *neu *gene, and cannot be replaced by non-specific stimulation with SINCP-βgal.

### neu-Specific IgG in the serum of prime–boost-vaccinated mice

**Figure 5:**
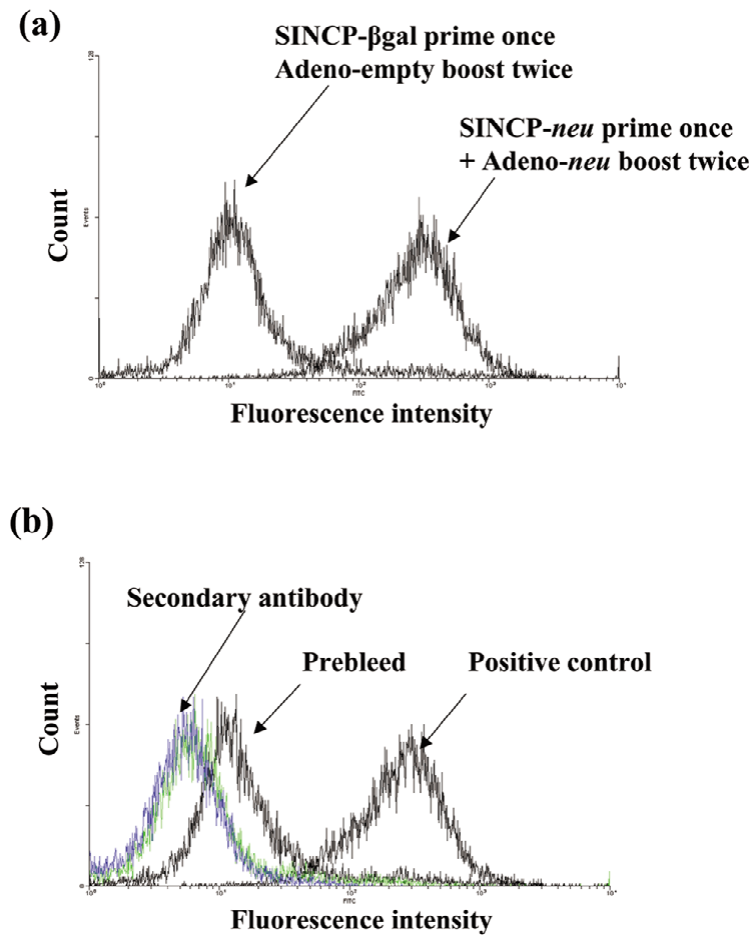
Flow cytometric analysis using A2L2 cells of serum from mice vaccinated with SINCP and Adeno. Naive mice were vaccinated with SINCP-*neu *(a plasmid containing Sindbis virus genes and the gene for rat HER2/*neu*) as the prime and twice, at 2-week intervals, with Adeno-*neu *(an E1,E2a-deleted adenovirus also containing the gene for rat HER2/*neu*) as the boost; or they were vaccinated with SINCP-βgal as the prime and then twice, at 2-week intervals, with Adeno-empty (adenovirus lacking an inserted gene) as the boost. **(a) **Immune serum collected 2 weeks after the second Adeno vaccination was diluted 1:100 and analyzed by flow cytometry. **(b) **A2L2 cells treated with fluorescein-isothiocyanate-labeled goat antimouse IgG (secondary antibody) and serum collected before vaccination (prebleed) served as the negative controls, and A2L2 cells treated with a commercial polyclonal antibody against p185 served as the positive control.

We evaluated the effectiveness of prime–boost vaccination in an *in vitro *assay. Naive mice were primed by vaccination with SINCP-*neu *and boosted by vaccination twice with Adeno-*neu*, or they were primed by vaccination with SINCP-βgal and boosted by vaccination twice with Adeno-empty [48]. The presence of the *neu *gene in both the prime and the boost resulted in a strong IgG response to the A2L2 cells (Fig. 5a). The IgG response from vaccination with SINCP-βgal followed by Adeno-empty (Fig. 5a) was nearly identical to the IgG level in serum collected from the mice before vaccination (Fig. 5b). These observations indicated that prime–boost vaccination produces strong humoral immunity.

### Induction of IFNγ-producing cells in the spleens of prime–boost-vaccinated mice

**Figure 6:**
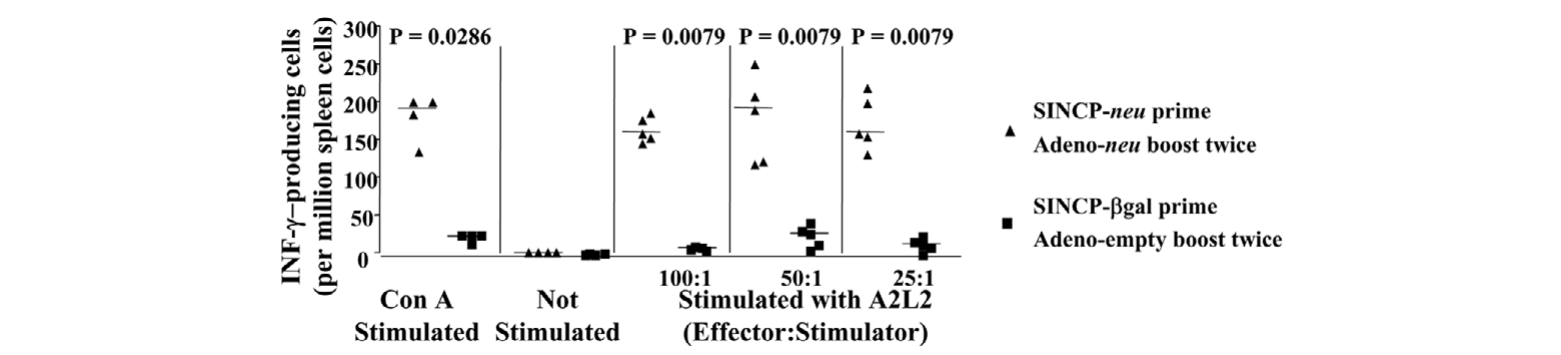
IFNγ ELISPOT analysis of spleen cells from mice vaccinated with SINCP (prime) and Adeno (boost). Naive mice were vaccinated with SINCP-*neu *(a plasmid containing Sindbis virus genes and the gene for rat HER2/*neu*) and then twice, at 2-week intervals, with Adeno-*neu *(an E1,E2a-deleted adenovirus also containing the gene for rat HER2/*neu*). Spleen cells were collected 2 weeks after the second Adeno vaccination and were cultured overnight with 5 μg/ml concanavalin A, without stimulation, or with A2L2 cells at three different effector:stimulator ratios. The number of IFNγ-secreting cells was determined using a commercial ELISPOT procedure and antibody pair. Horizontal bars indicate means. IFNγ, interferon γ.

To determine whether prime–boost vaccination produced *neu*-specific T lymphocytes, we used an IFNγ ELISPOT assay to compare spleen cells of mice vaccinated with SINCP-*neu *as the prime and Adeno-*neu *as the boost with spleen cells of mice vaccinated with SINCP-βgal as the prime and Adeno-empty as the boost. Spleen cells from vaccinated mice were co-cultured overnight with A2L2 cells, and the next day the number of cells producing IFNγ was determined. Vaccination with SINCP-*neu *followed by Adeno-*neu *resulted in a mean of more than 150 IFNγ-producing cells per million spleen cells at all ratios of effector (spleen cell) to stimulator (A2L2 cells) cells, whereas vaccination with SINCP-βgal followed by Adeno-empty resulted in a mean of fewer than 20 IFNγ-producing cells per million spleen cells (Fig. 6). As a positive control and possibly as a measure, of a maximum response, spleen cells were stimulated with 5 μg/ml Con A, which resulted in a mean that was comparable to the means obtained with *neu *in both the prime and the boost. That the presence of *neu *in both the prime and the boost resulted in IFNγ production after co-culture with A2L2 cells and that this effect was not seen with vaccination with SINCP-βgal followed by Adeno-empty clearly demonstrated that prime–boost vaccination resulted in antigen-specific induction of cellular immunity.

## Discussion

In this report, we showed that vaccination with a plasmid or adenovirus vaccine containing the gene for *neu *protected mice from challenge with breast cancer cells. In the mammary fat pad model, a single vaccination with either SINCP-*neu *or Adeno-*neu *was effective. In the experimental lung metastasis model, a single vaccination with Adeno-*neu *was effective, and the SINCP-*neu *vaccine was effective when delivered three times at 2-week intervals.

In our study, serum from mice vaccinated with SINCP-*neu *or Adeno-*neu *contained high levels of IgG that reacted with A2L2 cells, whereas serum from mice vaccinated with SINCP-βgal or Adeno-empty was at background levels. We previously showed that immune serum from gene-vaccinated mice does not react with the parental cell line (66.3) that was transfected with the *neu *gene to create A2L2 [29]. The level of immunoflourescence resulting from a single vaccination with Adeno-*neu *was one log greater than that resulting from three vaccinations with SINCP-*neu*. Taken together with the results described in the previous paragraph for the experimental metastasis model, this finding indicates that at these doses and this schedule, Adeno-*neu *is the more immunogenic vaccine. Of course, there is no way to accurately compare a plasmid vaccine with a viral vaccine except in relative terms.

Although both SINCP-*neu *and Adeno-*neu *produced robust protection in two tumor-prevention models, both gene vaccines were ineffective when used after A2L2 cells were injected into the mammary fat pad or i.v., even when vaccination was begun only 2 days after tumor cell injection and vaccination was repeated three times for SINCP-*neu*. Adeno-*neu *was injected only once in these therapeutic models. Because many publications have described the effectiveness of prime–boost vaccination in which the prime is a plasmid gene vaccine and the boost is a viral gene vaccine, and have shown that priming with a plasmid and boosting with a virus is more effective than the opposite [43,44,47], we tested this strategy in our two therapeutic tumor models.

In our experimental metastasis model, priming with SINCP-*neu *followed by boosting twice with Adeno-*neu *prolonged the lives of mice compared with prime–boost vaccination with SINCP-βgal and Adeno-empty. This effect was seen only in the experimental metastasis model and not in the experimental mammary fat pad model, and no other prime–boost combination worked. Although this combination of SINCP-*neu *and Adeno-*neu *was able to increase the survival rate, all mice eventually succumbed to tumor growth and were killed because of moribundity. We also found that prime–boost vaccination results in antigen-specific induction of both cellular and humoral immunity. A question that cannot be answered by our findings is the relative role of cellular immunity versus humoral immunity in the *in vitro *antitumor effect.

With the mandate to move promising laboratory findings to the clinic, it is most important to evaluate the implications of these findings with regard to treatment of patients. A gene vaccine against HER2/*neu *could be effective for patients with a HER2/*neu*-expressing tumor but could not be rationally used to manage a HER2/*neu*-negative tumor. Many types of HER2/*neu*-expressing tumors are noteworthy for their aggressive growth and high metastatic potential [50]. Our data indicate that combined vaccination in a prime–boost schedule may be the most likely to produce a clinical effect. However, these vaccines require phase I toxicity testing individually before they could be evaluated in a prime–boost protocol. A phase I/II study may yield valuable information regarding increased survival rates and induction of cellular and humoral immunity as measured by *in vitro *assays. Testing of either SINCP-*neu *or Adeno-*neu *in patients will require the production of clinical-grade material, which could be less of a hurdle for a plasmid than for a virus. Nonetheless, our data identify two promising gene vaccines against HER2/*neu*, an antigen associated with aggressive tumor growth and metastasis.

## Conclusion

We demonstrated that in mice, vaccination with a plasmid gene vaccine and an adenovirus gene vaccine, each containing the gene for HER2/*neu*, prevented growth of a HER2/*neu*-expressing breast cancer cell line injected into the mammary fat pad or i.v. The gene vaccines were not effective individually in therapeutic vaccine models in which vaccination took place after tumor cells were injected. However, sequential administration of the vaccines in a prime–boost protocol was therapeutically effective when the tumor cells were injected i.v. The vaccines induced high levels of both cellular and humoral immunity as determined by *in vitro *assessment. Clinical evaluation of these vaccines, particularly when used sequentially, is justified.

## Abbreviations

Adeno = adenovirus; Con A = concanavalin A; EDTA = ethylenediaminetetraacetic acid; ELISPOT = enzyme-linked immunospot; FITC = fluorescein isothiocyanate; i.m. = intramuscular or intramuscularly; i.v. = intravenous or intravenously; IgG = immunoglobulin G; IFNγ = interferon γ ; PBS = phosphate-buffered saline.

## Competing interests

The authors declare that they have no competing interests.

## Authors' contributions

The authors' contributions to this research are reflected in the order shown. XW performed all molecular biology in Dr. Lachman's laboratory, all animal experiments and all laboratory analysis; J-PW was responsible for cell culture; X-MR prepared and purified the Adeno-*neu*; JEP supervised the initial animal experimentation and developed the tumor model; HSZ performed all molecular biology associated with the adenovirus work and LBL supervised all aspects of the research and wrote the original manuscript. All authors read and approved the final manuscript.
